# Multiple impact pathways of the 2015–2016 El Niño in coastal Kenya

**DOI:** 10.1007/s13280-020-01321-z

**Published:** 2020-03-09

**Authors:** Matt Fortnam, Molly Atkins, Katrina Brown, Tomas Chaigneau, Ankje Frouws, Kemyline Gwaro, Mark Huxham, James Kairo, Amon Kimeli, Bernard Kirui, Katy Sheen

**Affiliations:** 1 Department of Geography, College of Life and Environmental Sciences, University of Exeter, Amory Building, Rennes Drive, Exeter, EX4 4RJ UK; 2 Environment and Sustainability Institute, University of Exeter, Penryn Campus, Cornwall, TR10 9FE UK; 3 Centre for Marine Ecosystem Research, Edith Cowan University, 270 Joondalup Drive, Perth, WA 6027 Australia; 4 Department of Natural Resources, Faculty of Environment and Natural Resources Development, Egerton University, P.O. Box 536, Egerton, 20115 Kenya; 5 School of Applied Sciences, Edinburgh Napier University, Edinburgh, EH11 4BN Scotland, UK; 6 Department of Oceanography and Hydrography, Kenya Marine and Fisheries Research Institute, P.O. Box 81651, Mombasa, 80100 Kenya; 7 Leibniz Centre for Tropical Marine Research (ZMT) GmBH, Fahrenheitstr. 6, 28359 Bremen, Germany; 8 International Development Department, School of Government, Muirhead Tower, University of Birmingham, Edgbaston, Birmingham, B15 2TT UK

**Keywords:** Climate variability, Coastal social-ecological systems, El Niño, Resilience, Vulnerability

## Abstract

The 2015–2016 El Niño had large impacts globally. The effects were not as great as anticipated in Kenya, however, leading some commentators to call it a ‘non-event’. Our study uses a novel combination of participatory Climate Vulnerability and Capacity Analysis tools, and new and existing social and biophysical data, to analyse vulnerability to, and the multidimensional impacts of, the 2015–2016 El Niño episode in southern coastal Kenya. Using a social-ecological systems lens and a unique dataset, our study reveals impacts overlooked by conventional analysis. We show how El Niño stressors interact with and amplify existing vulnerabilities to differentially impact local ecosystems and people. The policy significance of this finding is that the development of specific national capacities to deal with El Niño events is insufficient; it will be necessary to also address local vulnerabilities to everyday and recurrent stressors and shocks to build resilience to the effects of El Niño and other extremes in climate and weather.

**Electronic supplementary material:**

The online version of this article (10.1007/s13280-020-01321-z) contains supplementary material, which is available to authorized users.

## Introduction

The El Niño Southern Oscillation (ENSO) is the most significant driver of global inter-annual climate variability. The strongest El Niño events have far-reaching consequences for worldwide temperature and rainfall patterns, causing extreme droughts and flooding, mass coral reef bleaching, and major impacts on economies, health and food security (Glantz 2001; Field et al. 2014). In 2015–2016, one of the strongest El Niño episodes on record affected an estimated 60 million people globally (UN 2015). Climate change may make such ‘super El Niño’ events more common (Cai et al. 2014). Thus, there is an urgent need to understand what makes people vulnerable, and how societies and ecosystems can be made more resilient, to the effects of El Niño.

Past research on El Niño impacts has studied its influence on regional climates (see Dong et al. 2016), its relationship with disease incidence (Kovats et al. 2003), vulnerability to mass coral bleaching (e.g. McClanahan et al. 2009; Cinner et al. 2013), changes in fish abundance and distribution (e.g. Mysak 1986; Glantz 2005) or its impact on national economies, sectors or food security (Glantz 2001). These studies focused on single stressors (e.g. drought or floods) and/or specific impacts (e.g. disease incidence or coral bleaching) on either people or the environment. Indeed, conventional analysis of El Niño impacts tends to rely on studying direct linear pathways from a single physical signal (e.g. climate and sea temperature perturbations) to biophysical stressor (e.g. flooding or coral bleaching) to direct impacts (e.g. loss of life or property).

Table 1 shows that studies of El Niño events in Kenya, the site of this study, also investigated single-stressor impact pathways, and particularly the impact of the 1997-98 El Niño event, which brought intense rainfall and flooding to Kenya, destroying infrastructure and property, displacing thousands of people and resulting in disease outbreaks (see Dong et al. 2016; Hameed et al. 2016). Since this event, significant investment has been made in early warning systems, improving the preparedness of key economic sectors and humanitarian response in Kenya (IFRC 2014; Rono-Bett 2018).

**Table 1:** Known impacts of El Niño events on Kenya

Domain	Impacts	References
Climate variability and extremes	Correlations between ENSO conditions and monthly and seasonal rainfall patterns in East Africa have been observed. Kenya tends to experience increased rainfall during the rainy seasons and is prone to flooding during El Niño episodes. Heavy rainfall events caused landslides in 1997–1998 in several areas of Kenya	Ngecu and Mathu (1999), Li et al. (2016), Mutemi (2003), Muthama et al. (2014)
Coastal ecosystems	High sea-surface temperatures associated with El Niño events can result in coral bleaching and mortality. In 1997–1998, there was up to 95% coral mortality in Kenya, with variable rates of recovery. Sedimentation in mangroves by floodwaters caused forest dieback in many areas along the Kenyan coast	Westmacott et al. (2000), Baker et al. (2008), M’rabu Jenoh (2009), Wieczkowski (2009)
Disease incidence	There is strong evidence that El Niño events can increase cholera risk and promote malaria epidemics. In Kenya, Rift Valley Fever (RVF), malaria, cholera and dysentery have been linked to El Niño-related flooding. The economic cost of RVF in East Africa exceeded 60 million USD during the 2006–2007 El Niño event	Anyamba et al. (2001, 2009), Linthicum et al. (1999), Woods et al. (2002), Little (2009)
Economy and coastal livelihoods	Kenya’s predominantly agricultural economy is vulnerable to fluctuations in rainfall caused by ENSO. The national cost of the 1997 event to the Kenyan economy was estimated at one billion USD. Extreme events, such as storms and floods, destroy livelihood infrastructure, such as fish landing sites, farm storage and transportation routes to market, and damage assets such as fishing boats and gear. Although impacts are difficult to discern and not immediate, coral bleaching is likely to have long-term negative effects on Kenyan reef fisheries and the amenity value of the reefs for dive and snorkelling tourism. Mangrove destruction reduces coastal fisheries and removes sources of forest products widely used by local people	Ngecu and Mathu (1999), Westmacott et al. (2000), McClanahan et al. (2002), Grandcourt and Cesar (2003), Pratchett et al. (2008)

In this work, we adopted a social-ecological systems approach to understand the impacts of the 2015–2016 El Niño event in coastal Kenya. The El Niño teleconnection with regional climatic processes resulted in the displacement of rainfall further south and west in East Africa (Anyamba et al. 2018; Siderius et al. 2018), meaning it was considered nationally in Kenya to be a non-event (Development Initiatives 2017). We utilised multiple social and ecological methods and data sources that generated findings on the impacts of El Niño on a social-ecological system in Vanga, an area located in the southern Kenyan coastal county of Kwale. In the results, we analyse the ecological and human impacts of, and response to, the event, and how these impacts are linked to system vulnerabilities. We discuss how a social-ecological systems approach to understanding vulnerability helps to explain impact pathways as complex and dynamic rather than linear. Thus, El Niño events with weak national effects can still mean disaster for some people and places due to interactions with other stressors and local sensitivities and capacities. This has significant implications for how El Niño, and by extension climate change, is analysed and intervened with because it shifts the focus from the ‘event’ itself and, therefore, the policy preoccupation with early warning systems, to the need for addressing local vulnerabilities and for building social-ecological resilience in the context of local needs.

## Theoretical framework

While there are diverse conceptualisations of vulnerability, we adopted a recent approach that considers coupled human-environment (or social-ecological) systems to capture the complex processes and interlinkages that determine vulnerability (Turner et al. 2003; Bennett et al. 2016). While sub-systems (e.g. a coastal fishery), units (e.g. a household or mangrove tree) and governance systems (e.g. institutions) can be analysed separately, they interact in complex social-ecological systems. We adopted the common characterisation of vulnerability as the interaction of exposure (to shocks and long-term change), sensitivity (the susceptibility of the social-ecological system to harm) and resilience (the capacity of the system to buffer, cope, respond and adapt to shocks and long-term change) (Turner et al. 2003).

Assessing the vulnerability of a social-ecological system is inherently interdisciplinary, requiring assessment of ecological and social aspects, and the integration of quantitative and qualitative social and physical science methods. We drew upon multiple sources of primary and secondary biophysical and social data collected both purposefully for this research (between October 2016 and April 2017) and during two research projects, SPACES^1^ and CESEA^2^ (2013–2016) to explain the impacts of El Niño 2015–2016 in our study site of Vanga.

## Materials and methods

### Study site

We collected data at national and county levels to capture cross-scale drivers, but the primary focus was at the local level to understand vulnerability of a social-ecological system that could be practically studied. Our case study encompasses three villages—Jasini, Jimbo, Vichigini—and Vanga town, located in Vanga sub-location, a rural administrative unit in Kwale County on the south coast of Kenya. We selected this study site for two reasons: (i) it was known to have experienced a severe flood during the 2015–2016 El Niño in April 2016, enabling us to explore a shock event; and (ii) the research team has been studying the site and surrounding area for several years, providing existing datasets to draw upon for contextual information and longitudinal social and ecological data.

**Fig. 1:**
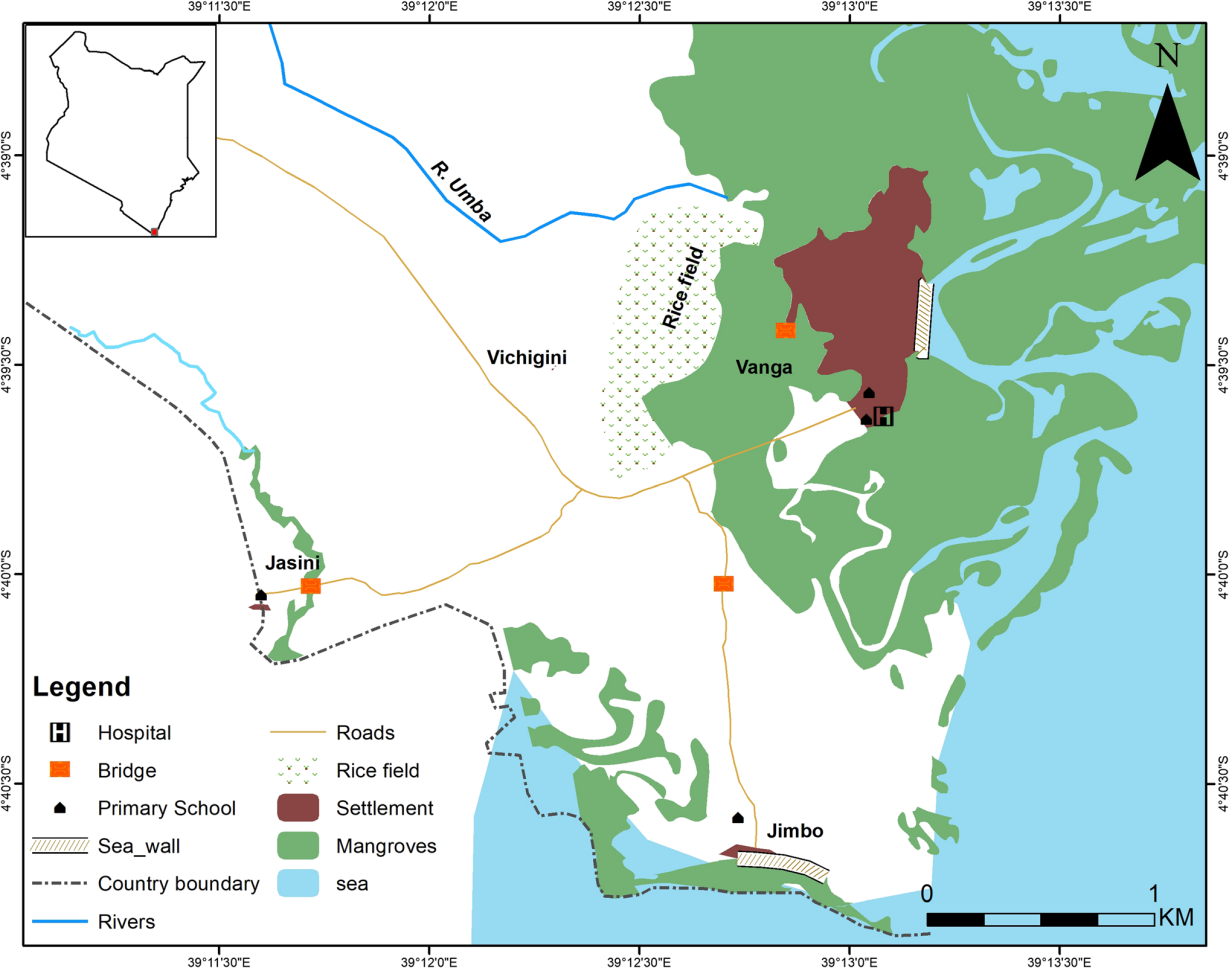
Map of Vanga sub-location. Map credit: Fredrick Mungai Mburu

Vanga is located at the mouth of the river Umba. The river drains its catchment in northeast Tanzania (Fig. 1) from its source in the Usambara Mountains, before crossing the Kenyan-Tanzanian border and flowing into the Indian Ocean through an extensive mangrove system at Vanga. Southern Kenya and north Tanzania have two rainy seasons: the long rains (~ March to May) and the short rains (~ October to December). During these rainy seasons, the river Umba floods into the mangrove complex and areas now cultivated or settled.

The population is estimated to be 6500 (Fortnam et al. 2019), comprising of three main ethnicities – Digo, Duruma and Kamba. Unlike the mixed faith settlements further north on the Kenyan coast, almost all residents in Vanga sub-location are Muslim. Their main occupations are fishing, farming, trading and construction. The fishing industry is the most important sector in Vanga; about half of household’s livelihoods are dependent on it and Vanga is the main fisheries landing site south of Mombasa (HHS1, Appendix S1). There are also many smallholder farmers, who commonly grow rice and cassava on the fertile riverbanks of the Umba and/or engage in livestock husbandry. Overall, Kwale County and Vanga perform poorly on available wellbeing indicators compared to national averages (Table 2), and over 30% of households in Vanga do not meet their basic needs for water, sanitation, economic security or food security (SPACES 2017; Chaigneau et al. 2019).

**Table 2:** Comparison of wellbeing indicators for Vanga sub-location, Kwale county and Kenya

	National	Kwale	Vanga
% food poor individuals	32	41.1	ND
% overall poor individuals	36.1	47.4	ND
Total household monthly expenditure	7811 ksh (75 USD)	6470 ksh (62 USD)	ND
% individuals not educated	25.2	38.6	40.5
% individuals primary level education	52	51	52.4
% individuals with secondary level education or more	22.8	10.4	7.1

With the exception of primary school education, indicators for Kwale and Vanga perform worse than national averages. Source: KNBS (2018a, b)

Vanga Bay supports 2351 ha of mangrove forest, the largest area of mangroves on the south coast of Kenya. Whilst the forest has a long history of human use, especially for firewood and poles for construction, it is not as degraded as mangrove forests elsewhere on the south coast (Huxham et al. 2015). There are extensive areas of seagrass in the Bay, which are likely to be important for fisheries' provision, but heavily impacted by human activity, particularly seine netting (personal observation). Kenya’s fringing coral reef stretches from Vanga until Malindi, north of Mombasa, and is a critical habitat that supports the small-scale fisheries of Vanga.

Appendix S1 summarises the methods used in the research and the data that inform the analysis below. We designed the methods to record both the impact of the 2015–2016 El Niño event and the vulnerability of Vanga to a range of long-term stresses and shocks experienced today and historically. This enabled us to explore how general system vulnerabilities determined the impact of a specific El Niño event on a social-ecological system.

To understand social vulnerability at the community level, including the range of stressors and shocks faced and social sensitivities and capacities, we adapted Climate Vulnerability and Capacity Analysis (CVCA) tools (Daze et al. 2009). At a focus group (referred to as FG1), 12 participants, representing a range of social groups in Vanga, used these tools to identify: (i) risks faced by the community; (ii) who and what is sensitive to them; and (iii) capacities for coping and adaptation. Such bottom-up participatory methods are increasingly used to identify local level determinants of vulnerability based on people’s experiences rather than assuming them beforehand (e.g. Ford and Smit 2004; McCubbin et al. 2015). Secondary and digitised data on socio-economic, demographic and environmental change were collected to substantiate longer-term trends and stressors on the community.

The impact of the 2015–2016 El Niño event on precipitation was assessed in comparison to average seasonal precipitation and average seasonal precipitation during strong to very strong El Niño and La Niña events using GPCC data at two relevant locations (4.25° S, 38.75° E and 4.75° S, 38.75° E) in the Umba drainage region. Strong El Niño and La Niña events were classified, using an Oceanic Niño index in the 3.4 region,^3^ averaged over December, January and February, as being greater or less than 1.5, respectively. Relevant rainfall gauge data were only available at Lunga-Lunga, 15 km upstream of Vanga, between 1960 and 2002; however, these data strongly correlated (coefficient *r* = 0.80, *p*< 0.001) with the GPCC rainfall data for this period at 4.75° S, 38.75° E, giving confidence in utilising the product for localised rainfall estimates beyond 2002. The impacts on ecosystems were assessed using longitudinal data collected on mangroves, sea grasses and coral reefs. The 1998 El Niño event caused large-scale die-back in some mangrove areas in Kenya due to the substantial influx of sediments, carried by heavy rainfall on eroding coastal catchments (Bosire et al. 2014). This documented vulnerability to sedimentation informed our decision to measure sediment dynamics and the indirect signal of mangrove tree growth. The nearest site with such monitoring data available is Gazi Bay, 40 km to the north of Vanga. For coral reefs, bleaching surveys were undertaken at 24 sites in Kenya using a rapid assessment field method; the closest survey site to Vanga was Kisite, 13 km offshore from Vanga (McClanahan and Darling 2016). These surveys were part of an ongoing reef-monitoring programme, which does not collect data on the most proximate reefs to Vanga, but the results provide an indicative measure of coral bleaching on reefs that support the Vanga fisheries.

Direct and indirect social impacts and responses to the 2015–2016 El Niño event were recorded during the forementioned CVCA focus group, three household surveys (HHS1, HHS2, HHS3) and key informant interviews (KIIs) in November to December 2016. Thus, immediate impacts were recorded based on the recollections of research participants, about 6 months after the El Niño episode ended. At the workshop, participants discussed the El Niño event in relation to each workshop activity (Appendix S1). For example, after creating a seasonal calendar of a typical year, participants discussed how the seasons differed during the El Niño episode. A major flood in Vanga in April 2016 was experienced during the El Niño period. A census household survey (HHS1; *n* = 102) was undertaken in the three study villages to understand impacts on wellbeing and responses to the flood event. To capture impacts on a marginalized group, an additional survey (HHS2; *n* = 20) of female-headed households living in the flood-prone villages of Jimbo and Jasini, and Vanga town was conducted to understand how the multiple effects of the flood manifested and interacted to affect household wellbeing of a vulnerable social group. Local school attendance and hospital admission records was digitised and analysed to quantify health and education impacts from the flood. A further survey (HHS3) of 15 households (previously surveyed by the SPACES project) investigated household impacts, preparations and perceptions of El Niño beyond the flood event.

## Results

### Biophysical impacts and exposures

**Fig. 2:**
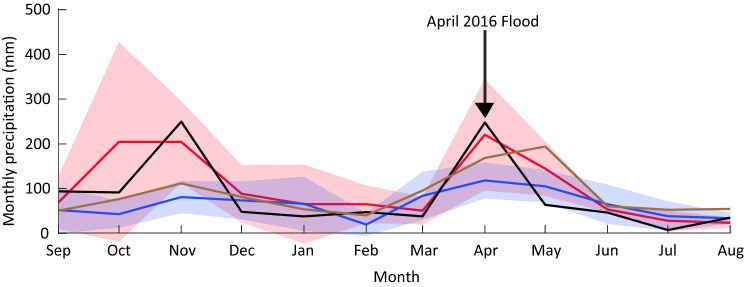
Seasonal rainfall in lower Umba drainage basin as mean (1891-2016; brown line), during very strong El Niño events (ONI for December, January and February (DJF) > 1.5; red line), and very strong La Nina events (ENSO 3.4 index for DJF < − 1.5; blue line). Shaded regions show 5–95% confidence interval (computed using $t\sigma /\sqrt {\left( {n - 1} \right)}$, where $t$ is the one-sided *t* distribution value, $\sigma$, the standard deviation of the sampling distribution, and $\left( {n - 1} \right) = 4,$ the degrees of freedom). The data show that rainfall during very strong El Niño events is significantly higher than during very strong La Nina events in November, and above average during the October–November and April rains. The seasonal rainfall anomalies in the lower Umba basin between September 2015 and August 2016, associated with the 2015/16 El Niño event (black line) align with this pattern. Data also show that there was below-average rainfall before and following the 2016 April extreme rains

Globally, the 2015–2016 El Niño episode was one of the strongest ever recorded (L’Heureux et al. 2016) but, in Kenya, El Niño was widely considered to be a ‘non-event’, whereby impacts were experienced but to a lesser magnitude than was predicted, likely due to rainfall being displaced south and west (Development Initiatives 2017; Anyamba et al. 2018; Siderius et al. 2018). Nevertheless, precipitation data shows that the lower Umba river drainage basin received above long-term mean rainfall (1891–2016) during both the short (November 2015) and long (April 2016) rainy seasons (Fig. 2). While it was beyond the scope of this study to attribute this heavy rainfall with El Niño and associated teleconnections emphatically, these patterns align with those associated with past strong El Niño events, as shown in Fig. 2, indicating a possible influence of ENSO.

Figure 2 also shows that precipitation deviated most from its long-term mean in the 2015 short rains, and peak rainfall was similar across both rainy seasons. However, it was in April 2016, during the short rains, that the river Umba burst its banks, flooding many villages in Vanga sub-location. Because GPCC rainfall data is provided as monthly rather than daily means and with a lack of localised rain gauge or river drainage data for 2016, it was not possible to analyse whether the April 2016 flooding was due to differences in rainfall intensity, the dry period prior to April increasing surface run-off, and/or more localised precipitation distributions and drainage basin processes in the Umba catchment.

**Fig. 3:**
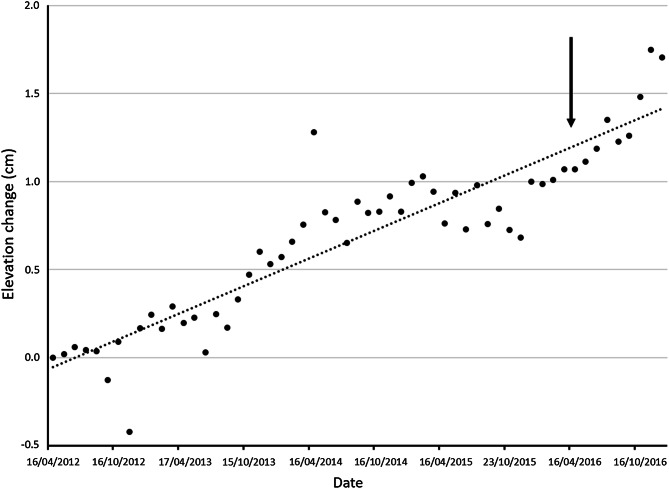
Surface elevation change measured in ten mangrove plots at Gazi Bay (4° 25’ S, and 39° 30’ E), using rods (see Lang’at et al. 2014 for full details of initial design). The arrow marks April 2016, the period when flooding was experienced in Vanga; there is no evidence of enhanced sedimentation. The fitted line shows elevation change of 3.1 mm year^−1^ in this healthy *Rhizophora mucronata* forest, showing a robust response to sea level rise

While heavy rainfall during past El Niño events had significant impacts on mangroves in Kenya because of sedimentation (Bosire et al. 2014), we found no evidence of enhanced sedimentation (as manifested in surface elevation rise) and no evidence of changed growth rates of the monitored mangrove plots at Gazi (Fig. 3), and are not aware anecdotally of major reported impacts on mangroves. Similarly, there was no evidence of enhanced sedimentation in the monitored seagrass plots at Gazi, and Landsat satellite images show that mean turbidity in Vanga Bay during the long rains of 2016 was not significantly different to the same period in 2015. However, we recorded contradictory evidence during focus groups (1 and 2) that water visibility for spear fishers became poor because of sediment run-off, which may be due to the relatively coarse temporal and spatial resolution available from Landsat satellite data.

The CCVA showed that flooding is regularly experienced in Vanga. Even if there is little rainfall in Vanga itself, heavy rain upstream in the upper Umba catchment in Tanzania can cause the area to flood. The river flow of the Umba has high seasonal and inter-annual fluctuations due to variance in the strength and timing of the rainy seasons and climate variability (Fig. 2 shows the average effects of strong ENSO events on seasonal precipitation). Land-use changes from deforestation and cultivation in the catchment have also increased river flow rates (Scheren et al. 2016). In addition to riverine flooding, the seaward area of Vanga town and Jimbo village are exposed to spring tide flooding. Especially severe flooding is experienced when spring tides coincide with the Umba flooding (FG1, key informant). In other words, the location of Vanga in a mangrove complex at the mouth of a major river makes it highly exposed to riverine and tidal flooding (see map, Fig. 1). However, when CVCA participants developed a historical timeline of disaster events in Vanga they ranked the flooding in April 2016 as the fifth worst disaster event in their collective memory, with floods associated with El Niño in 1997 ranked the second-worst event. This history and precipitation data, therefore, demonstrates how El Niño events amplify existing flood risks and effects, rather than causing them.

In addition to rainfall anomalies, McClanahan (2017) found that the temperature of coastal waters in Kenya and Tanzania deviated significantly from its summer mean of 28.1 °C during the 2015–2016 El Niño event. Sea-surface temperatures peaked at 30.9–31.5 °C in March 2016 and persisted above their mean for 65 days in Mombasa, the closest data point to Vanga. The thermal anomaly during the 1997–1998 El Niño event peaked at a similar temperature of 30.9 °C, and the cumulative degrees above mean summer temperature (degree days) in both 1998 and 2016 was ~ 85° days, suggesting the thermal stress on coral reefs in Kenya was similar in both events. The surveys of coral reef colonies’ estimated bleaching response during 2016 using a 7-category scale – scaled from normal colour to pale – and estimated percentages of surface area bleached or recently killed. The results showed that bleaching effects were less severe in 2016 than 1998. The proportion of bleached colonies was 60% in 2016 compared to 96% in 1998 (McClanahan 2017); at the closest survey site to Vanga, about 35-50% of colonies were bleached in 2016.

### Human impacts

The April 2016 floods were the most tangible effect of the El Niño event for Vanga, according to participants. The household surveys (HHS1 and 2), focus groups (FG1 and 2) and interviews found that the floods impacted several aspects of household wellbeing (Table 3). None of the research participants identified impacts associated with the coral bleaching event. While there were likely to be effects from coral bleaching on reef fisheries, they are difficult to detect immediately because ecological interactions generate a lag time in the impact pathway (Graham et al. 2007).

**Table 3:** Reported impacts on several household wellbeing domains

Wellbeing domains affected	Impacts
Water	51% of respondents identified the contamination of drinking water supplies by flood water mixing with sewage as a community impact (FG1 and 2)
Sanitation	Latrines over-spilling caused sewage to mix with floodwaters (FG1 and 2) Unable to access mangroves to defecate, resulting in open defecation in community (HHS2)
Health	Decline in sanitary conditions increased the incidence of diarrhoea (FG1 and 2) 17.7% of households reported that at least one member experienced a waterborne disease (HHS1)
Education	Illness and loss of Jasini bridge prevented some pupils and teachers from attending school; 16.1%, 15.4% and 3.4% of Jasini, Vichigini and Jimbo households reported this impact (HHS1), respectively, while school attendance data in Fig. 6 suggests the proportion of pupils absent from school during the floods to be higher than that reported by households
Respect, autonomy and relations	Six female-headed households (out of 20 surveyed households) had houses damaged by floods and were forced to live with family or neighbours, which affected their social relations, reduced their sense of autonomy and/or made them feel shame (HHS2)
Shelter	22% houses destroyed; 25% damaged; 12% inundated (HHS1) 20% evacuated homes (HHS1)
Food	Food supply reduced due to combination of crop loss, reduced fish supply and disruption to food imports (FG1)
Economic security	
Agriculture	34.3% of households lost their crops, 10% of households lost livestock (HHS1)
Fisheries	Unable to export fish to larger markets in Mombasa due to inundation of main road (FG1, FG2, HHS1) Spoiling of fish because of wet conditions (FG1) and fishers from outlying villages were unable to sell their fish at Vanga fish market because of collapsed bridges (FG1, key informant, HHS2) Lost days at sea for spear fishers due to poor water visibility because of debris and high sediment loads (FG1)
Firewood trade	Flooded mangroves inaccessible for firewood harvesters (HHS2)
Trade	Unable to import or export goods because of flooding of main road (FG1)
Other	Working days were lost to sickness and income was spent on medication (FG1)

Data from household surveys (HHS1, 2), focus groups (FG1) and key informant interviews

The census survey (HHS1) found that over 80% of households in the three surveyed villages were affected by the floods and 47% of households experienced an economic security impact (Table 3). Out of the surveyed villages, riverside Vichegini had the largest proportion of households that lost their crops (61.5%) and suffered illness (38.5%), while Jasini had the largest proportion of houses damaged (61.3%). Key informants said that, overall, farmers living on the banks of Umba, mostly rice farmers, were the most impacted by the April floods. The female-headed household survey revealed that, despite the flood risks, people were attached to these riverside places of residence because of their social bonds within the communities, it being their ancestral homes, and proximity to the multiple wetland ecosystem services, such as fertile agricultural land, rich fisheries, and fuelwood and construction materials from mangroves, supported by the flood regime of the Umba (HHS2). These attachments to place, however, expose these social groups to the El Niño floods.

**Fig. 4:**
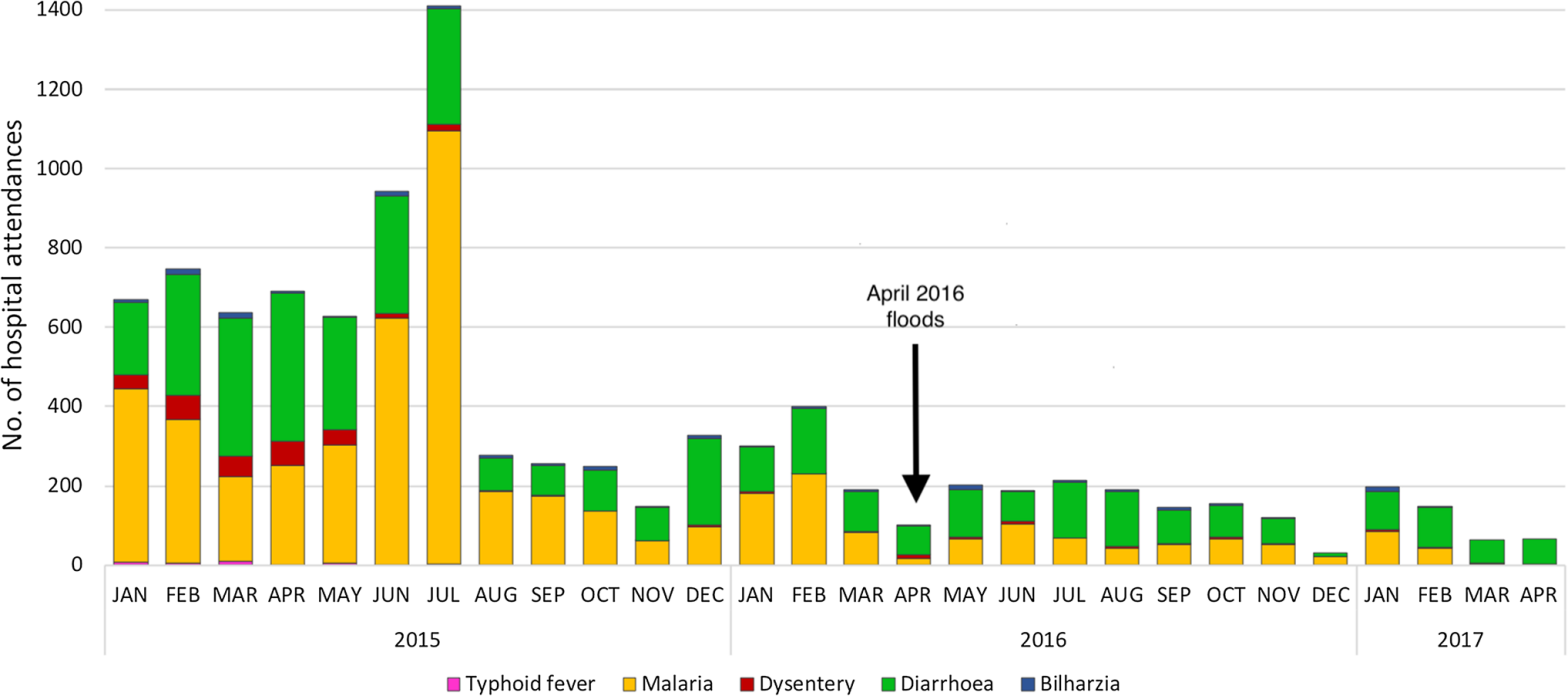
Number of attendees at Vanga Health Centre for waterborne diseases (January 2015 to April 2017). Admissions were low during April 2016 due to the inaccessibility of the health centre during the floods, according to KII. Source: Vanga Health Centre records

The impact of the floods on sanitary conditions and waterborne diseases (Table 3) is not surprising given that 46% of households in the three villages use an open latrine while 42% open defecate in the bush/mangroves, which were difficult to access during the floods, resulting in open defecation. Floodwaters, therefore, easily mixed with sewage, which contaminated drinking water sources; two-thirds of which are uncovered wells (HHS1). Likely due to these conditions, data from Vanga Health Centre shows prevalence of waterborne diseases the whole year round; 6–42% of monthly hospital attendance between January 2015 and April 2017 was associated with waterborne diseases. Disease incidence is elevated during the long rains regardless of whether El Niño conditions are present, and it was unclear from the data the extent to which the April 2016 floods increased waterborne disease incidence. Focus group (1) participants said that there were elevated rates of diarrhoea and waterborne disease compared to a typical rainy season, but data from Vanga Health Centre (Fig. 4) showed, conversely, lower hospital admissions due to waterborne disease during April 2016 floods and the entire rainy season compared to 2015 (data was not available to establish a historical mean for comparison). A doctor at Vanga Health Centre explained that:

“[The] 2016 flood effects were very severe, therefore, most people could not access the [health centre] facility due to broken bridges and submerged roads. In 2015, however, the turn up [at the health centre] was high since the floods were not severe and roads and bridges were all accessible during and even after floods. […In 2015], there was [also] an outbreak of diarrhoea in Tanzania [… that] spread to Vanga through immigrants who settled in Jasini [, elevating incidence rates…]

**Fig. 5:**
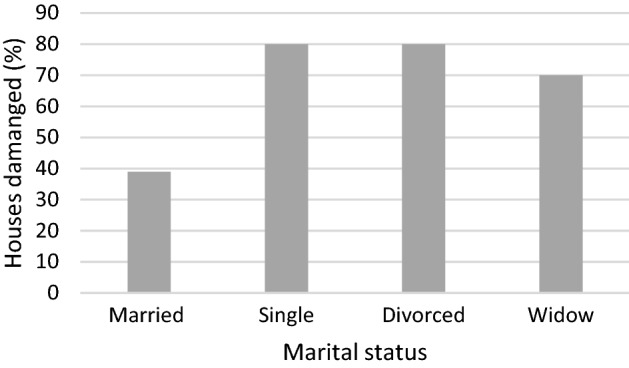
Percentage of houses damaged by floods by marital status. Data source: HHS1

Key informants identified those living in mud houses as especially affected by the April 2016 floods, and our household survey (1) recorded 68% of the houses damaged or destroyed by the floods as constructed of mud and thatch. These weakly constructed houses are most commonly inhabited by the very poor and female-headed households; this might explain why only 15% of the houses of male headed households were destroyed while 47% of all female-headed households lost their house during the floods. Indeed, fewer houses of married couples were destroyed compared to those of other martial statuses (Fig. 5). This points to the sensitivity of the least wealthy who are least able to afford robust shelter, and that the least wealthy are often marginalised groups like single women, widows and divorcees.

The disruption of trade and inaccessibility of schools and healthcare was linked to the submergence of the main road from Lungalunga to Vanga town for 3–4 days, and the destruction of bridges (Fig. 1) that link the outlying villages of Jasini and Jimbo to the main road (FG1, FG2, HHS1). Vanga is only accessible via the main road, which traverses a mangrove swamp before reaching Vanga. According to the CCVA, during seasonal river flooding or high tides, sections of the road are regularly submerged, and even heavy rains turn the road into mud. Value Chain Analyses of the Vanga fisheries showed that outside small-scale fish traders (*wachuuzi*) purchase fish at Vanga’s auction to sell in nearby markets, local large-scale fish dealers (*tajiri*) export fish by road to sell to fish shops as far as Mombasa, and large-scale octopus dealers collect octopus from Vanga to be processed and sold in Mombasa (Cheupe et al. 2017). Dependence on this road for trade with external markets (e.g. selling fish), makes the sub-location’s fisheries dependent economy vulnerable to shocks from flood events. In addition, while the impact of the April 2016 flood on fishing activity was minimal (a few lost days fishing), the fisheries sector was affected most by the inaccessibility of markets due to road disruption.

**Fig. 6:**
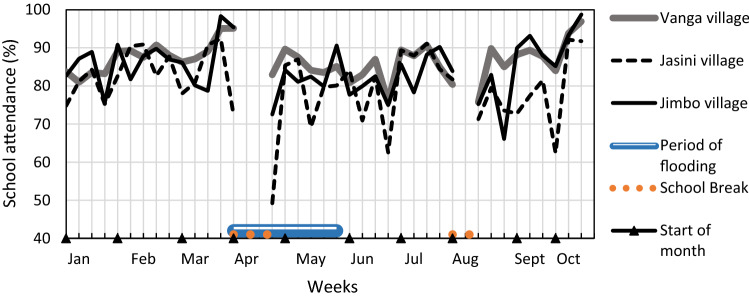
Weekly school attendance at Vanga primary school by students from Vanga town, Jimbo and Jasini. It shows how weekly school attendance at Vanga primary school by students from Vanga town, Jimbo and Jasini declined during and following the floods. Attendance is lower than normal after every school break because parents need to clear school fee debts before the children can begin the term. However, the decline in attendance after the April 2016 break is more pronounced than normal during the floods, especially amongst students from Jasini, who needed to be ferried across a treacherous, wide and fast flowing section of the river Umba to reach Vanga because of the collapsed bridge. Data source: School attendance records collected and digitised

The CVCA (FG1 and KIIs) identified perceptions of how the impacts of the flood event interacted. Decreased agricultural production and disruption to fisheries trade reduced the food and income of farmers and fishers, and regional declines in agricultural production and the disruption of the road network reduced food imports to the sub-location, which, together, raised the price of food. Households whose crops failed were, therefore, forced to purchase this relatively expensive food. Furthermore, if a member of a household contracted a waterborne disease, working days were lost to sickness and income was spent on medication. These interactive impacts manifested in hunger for 5.9% of households (HHS1), and increased expenditures, thus making it difficult for some families to pay school fees (FG, KII). While school attendance is normally lower at the beginning of term because parents need to clear school debts, there was a more pronounced drop in attendance during the 2016 floods, especially in children from Jasini, because the collapsed bridge made travel across the river dangerous (Fig. 6). Attendance recovered within a week, but the flood event had a short-term impact that added to an existing problem of children of poorer families missing school.

**Fig. 7:**
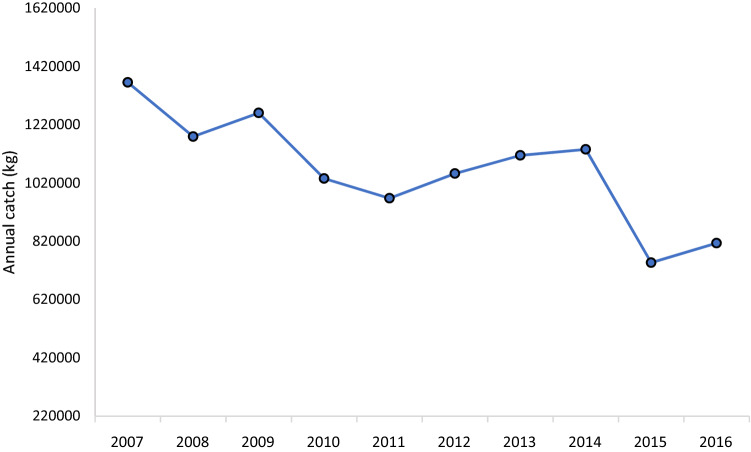
Total annual commercial fish catches at Vanga, taken from official data recorded by the Fisheries Department. Data cover all species caught, including crustaceans and cephalopods

Qualitative data from the female-headed household survey showed how for certain households the multiple impacts listed above can have cumulative impacts, as illustrated by the experience of this respondent who lost access to shelter, food and ecosystem services, and was unable to afford school fees:

‘The food that was stored in the house was destroyed during the flood. We did not have any food so we asked the neighbours to help but they could not help everyday so we often missed meals. One of my children left to go and live with a friend to help out … I am still living in a neighbours’ house and I feel like everything I do is going to annoy him.

I usually sell firewood, but during this time I could not go to the mangrove forest to collect firewood. The firewood needs to be dry. My children stopped going to school for 4–5 months because I am on my own and I could not do my business selling firewood, and there was no money to pay school fees.’ Yasmin

### Responses and capacities

Table 4 outlines responses to the flood event in Vanga by various social actors. At the national level, the Kenyan government convened an El Niño Multi-Sectoral Task Force under the Ministry of Interior, which compiled an El Niño Response Plan that outlines response strategies and resource requirements by sectoral ministries (NDOC 2015). However, government ministries only acted 1 month before the onset of rains when the Department of Meteorological Service forecasts became certain, despite other early warning system announcements about the El Niño event earlier in 2015; this delay was said to have weakened national preparedness (Rono-Bett 2018). At the county-level, upon receiving the warnings about the 2015–2016 El Niño, the Kwale County Stakeholder Forum convened to make humanitarian assistance preparations (KII). A community meeting (*baraza*) was hosted in Vanga by the local chief, the National Drought Management Authority, and the Kenyan Red Cross (KRC) to inform residents of the predicted El Niño event. Out of the 15 households surveyed specifically about the El Niño event (HHS3), seven did nothing in response to warnings, with the rest buying mosquito nets (3) staying away from risky areas such as riverbanks (3) or strengthening (2), or digging ditches (2) around, their house. It is not clear whether these reported responses may be part of annual preparations for the wet season regardless of El Niño warnings. Similarly, while half (51) of HHS1 respondents said they had received a forecast of heavy rains in April, 80% did nothing to prepare for floods (HHS1). The data did not explain why most did not respond.

**Table 4:** Responses to April 2016 flood by actor group

	Preparations	During	Recovery
Households (HHS1)	Nothing (80.4%) Moving to higher ground (3.9%) Repairing home (3.9%) Protecting home (e.g. with sand or clearing drainage channels) (2.9%)	Nothing (30.4%) Protecting the house against water (e.g. digging ditches or piling soil around the house) (21.6%) Evacuation (16.7%) Elevating cooking equipment above water level (11.8%) Living in inundated home and storing possessions in dry places (e.g. neighbours’ homes or roof) (4.9%)	Nothing (38.2%) Renovating homes (32.4%) Building new home (8.8%) Seeking help (6.9%) Return home (3.9%) Replanting crops (2.9%)
Community (FG1, KIIs)	Community meeting (*baraza*) about the El Niño event	Boats ferried or men carried people across floodwaters Assistance with humanitarian needs assessment	Temporary bridges constructed
County government (KIIs, FG1)	Kwale county stakeholder forum on the El Niño event	Humanitarian relief (food) Treatment of diseases at Vanga Health Centre Treatment of contaminated water sources	Donated seeds to farmers to replant crops Commissioned the construction of new bridge to Jasini
Kenya Red Cross (FG1, KIIs)	Organised El Niño *baraza*	Assessment of humanitarian needs Humanitarian relief (evacuation, shelter, emergency medical assistance)	

During the floods, community-based actions were limited to assisting KRC with humanitarian needs assessments. For female-headed households (HHS2), some evacuated their destroyed home to live with a neighbour, which affected their social relations, some diverted cash normally used to restock their business to buy food and pay school fees, and others sold household possessions to buy food: “I used what I had from the business, what I would have used to buy clothes in Mombasa, to buy food and pay for school fees”. County government support only reached 7% of surveyed households (HHS1), while KRC played a critical role in the distribution of relief items, such as food, shelter and medical assistance, according to CVCA participants.

The April 2016 flood demonstrated the general lack of capacity to respond to disasters and address flood risk in Vanga. During the CVCA, participants reported a few adaptations made to reduce flood risk. A seawall was constructed in 1970, and then strengthened and extended in 2005 by the World Bank, and an irrigation scheme was established to control floodwaters and supply water to rice fields. The community also said that they had sought to protect the mangroves by controlling cutting in recognition of the mangroves’ flood and storm regulation services. These adaptations have done little, however, to mitigate flood risk, with the KRC assisting flood victims every year:

“Every year when it floods, we know who will need assistance. For instance, we know that an old man living in an inundated house will need to be convinced to leave his home”

Vanga has no community organisations responsible for disaster risk reduction or response, and few external interventions have sought to build resilience to the floods (FG1). This may explain the limited community level preparations and responses to the El Niño event and the April 2016 flood event, and the dependence of households on their own resources and humanitarian assistance. Eight months after the flood only just over half (56%) of households had repaired their houses, and the homes of four female-headed households remained uninhabitable. This weak capacity was attributed to a lack of financial and physical resources to reconstruct again (HHS1). Moreover, Jasini residents, a year after the flood, remain dependent on a fee-charging, privately owned temporary bridge (HHS1).

### Interaction of effects with social-ecological system variability and long-term trends

The April 2016 floods coincided with one of Vanga’s most difficult periods of the year identified on the seasonal calendar at the CVCA focus group. Participants explained that the long rains is the key planting season; food availability is often low because stores from previous harvests are running out and they must wait for the new crop to mature. It is also the low season for small-scale fishers since strong southerly winds (the *Kusi* season) often makes the sea treacherous for small fishing vessels. The El Niño flooding, therefore, exacerbated a food insecure period of the year.

The heavy rains of April 2016 were also followed by very low rainfall in the lower Umba drainage basin between May to August (Fig. 2) and a severe drought across Kwale county and much of Kenya, with the short rains (October–December) failing, triggered by connections between La Niña and Indian Ocean Dipole conditions in 2016–2017 (Uhe et al. 2018). Farmers in Kwale were unable to receive a harvest from the short rain planting season, causing prolonged food insecurity and malnutrition in late 2016 (IFRC 2017). In Vanga, farmers planted new seeds to replace crops destroyed by the April floods, but the harvest failed because of the onset of drought (FG2, HHS1, HHS2).

It is normal for a drought to follow the long rains during the dry season (June to October) (CVCA). While inland areas are more prone to drought than Vanga (key informant), every year, farmers are faced with the dilemma of whether to plant early in the long rainy season and risk their crop being destroyed by heavy rains and floods or wait until later in the rainy season but risk their crop perishing from drought: ‘For the farmers, you only plant once a year, if you miss the season, it is difficult for the whole year. You have to wait until the next year.’ (FG1). Farmers’ planting strategies are further complicated by inter-annual climate variability caused by El Niño, La Niña and the Indian Ocean Dipole. Uhe et al (2018) showed that La Niña increased the likelihood of drought in Kenya in 2016–2017, and Fig. 2 shows that below-average rainfall is associated with strong La Niña events in the lower River Umba during the wet seasons, and that rainfall in July–August 2016 was below to in line with average precipitation for strong La Nina events. The extreme drought exacerbated the effects of the El Niño event in Vanga in 2016, creating challenges for food production in Kwale County. The sequence of ENSO events, therefore, had cumulative effects.

Long-term stressors may also influence the vulnerability of households to El Niño events. Overexploitation of the fisheries and degradation of coastal marine ecosystems through destructive fishing and mangrove cutting has resulted in a sustained decline in fisheries production (Fig. 7^4^). A key informant said that migration to the area was considered to place additional pressure on fisheries and mangroves; 10% of households surveyed had lived in the area for less than 5 years (HHS1). According to CVCA (FG1) participants, the decline in the fisheries has reduced fish available for subsistence and increased the price of fish at local markets, with effects on economic and food security. Given the dominance of the fisheries sector in the local economy, there was some evidence that these trends are eroding capacities to deal with floods. For example, a female head of household (HHS2) said that she was unable to rely on her son’s low fish catch to offset impacts of the flood on her charcoal business. School headmasters expressed that many families struggle to pay school fees because of poverty, which the decline in the areas’ fisheries sector is likely to be exacerbating; lost income and increased expenditures associated with floods further strains household budgets and their capacity to pay school fees. During the CCVA (FG1), most participants agreed that, in general, the wellbeing of residents was deteriorating due to combined effects of increasingly severe floods and the decline in fisheries resources. The pronounced decline in the fisheries, therefore, likely weakens capacities to cope with floods and other shocks associated with El Niño events.

## Discussion

This article presents a social-ecological systems analysis of the complex human–environment interactions and social processes that determine vulnerability to, and the impacts of, El Niño events in place. The 2015–2016 El Niño event interacted with several features of the social-ecological system in Vanga.

First, direct social and ecological impacts were amplified or intensified through system feedbacks and social and biophysical responses (Birkeland 2004, Cinner et al. 2011). For example, food insecurity was caused by feedbacks between climate extremes, coastal livelihood activities, income, and food affordability and availability—effects of El Niño events that were also observed in Alaska and Peru (Badjeck et al. 2010). Connectivity in social-ecological systems also means the effects of El Niño events are not isolated to a single sector or livelihood. Rather than being solely dependent on fishing or farming, households often engage in several activities (Porter et al. 2008); while this diversity is known to be a source of resilience to climate extremes (Forster et al. 2014), this study shows that, when there are impacts on several sectors such as in Vanga, there can be cumulative effects on the wellbeing of households. These interconnections can be hidden in a sectoral analysis of vulnerability to climate variability or change (Badjeck et al. 2010).

The findings also show that the impacts of El Niño events are socially and ecologically differentiated. Coral reefs, one of the most sensitive ecosystems to climate variability (Walther et al. 2002), bleached while no changes were detected in mangroves or seagrasses, and poor female-headed households and farmers, especially those living in weak traditional houses, experienced the most severe immediate impacts on their wellbeing. In Vanga, the experience of some female-headed households, who are known to be socially and economically marginalized in East Africa (Clark 1984; Bryceson 2002), exemplified the disempowering consequences of effects of responses to direct impacts, such as selling assets, diverting investments and losing autonomy. Coping strategies can thus amplify the impact of El Niño events.

The evidence from Vanga also shows that system variability and dynamics affects the severity of impacts from El Niño events. In Vanga, the El Niño event amplified seasonal flood risks and seasonal food and income insecurity, and occurred against a backdrop of historic extreme events and regional patterns of inter-annual climate variability. Vanga has experienced successive harvest failures, El Niño associated floods are often followed by La Nina associated droughts (Rojas et al. 2014), and the extent of coral bleaching in 2015–2016 was likely mitigated by sensitive colonies of coral having been killed during past El Niño episodes (McClanahan 2017). Moreover, long-term trends and drivers of change, such as fisheries declines in Vanga, can exacerbate vulnerability to climate extremes (Wilbanks and Kates 2010). This finding demonstrates the importance of understanding interactions amongst multiple stresses and shocks to assess where and who is vulnerable (O’Brien and Leichenko 2000). System time lags can also make some social and ecological impacts indiscernible (Graham et al. 2007), such as the impact of coral bleaching on the fisheries in Vanga. The likelihood of more frequent El Niño events and reduced recovery time between events because of climate change (Field et al. 2014) suggests these vulnerabilities will become further amplified in future.

Focusing on single-impact pathways, single sectors, and single events in time masks how vulnerability to, and the effects of, El Niño events are amplified or attenuated through system feedbacks, amplifying effects, variability and cross-scale dynamics. These social-ecological systems features generate complex and non-linear impact pathways, meaning that El Niño events considered ‘non-events’ nationally can still have severe impacts on certain places and people. This perspective shifts the policy imperative away from developing only specific capacities to also developing general capacities to mitigate the impacts of El Niño events. Specific capacities refer to tools and skills needed to effectively predict, prepare and respond to specific climatic threats, while general capacities refer to those gained from human development that enable people to deal with and adapt to climate variability and change (Folke et al. 2010). In Kenya, improved climate information, early warning systems and preparedness, and information sharing amongst state and non-state actors has improved national-level El Niño-specific capacities (IFRC 2014). However, at the local level, this study showed that national early warnings and weather forecasts are not heeded. The fact that Vanga, a flood-prone area, lacks community institutions to manage risks and depends on humanitarian assistance, supports claims that local disaster risk reduction capacities remain weak in Kenya despite the devolvement of responsibilities for disaster management to county and local levels under the new constitution (Rono-Bett 2018). The findings from this study also show that general capacities in Vanga are low due to development deficiencies in shelter, health, water and sanitation, inequalities (e.g. female-headed households), and weak institutions that make the population of Vanga vulnerable to a range of stresses and shocks. Thus, low general capacity means Vanga lacks the ability to address specific El Niño event risks. This suggests that, to build resilience to El Niño events and other climate extremes, policy needs to build mutually supporting specific and general capacities at multi-levels and across multiple scales.

## Conclusion

We applied a social-ecological systems approach to understand the vulnerability of a coastal social-ecological system in Kenya to El Niño events. Applying participatory vulnerability analysis tools explained local vulnerabilities, while ecological and other social methods provided a disaggregated analysis of household vulnerabilities and El Niño associated impacts. Combining data gave a much more nuanced picture of El Niño impacts and needs than relying on biophysical or social data, or quantitative or qualitative data, alone.

The article shows how vulnerability to El Niño events and other climate extremes reflects multiple interacting drivers, stressors and socio-economic and ecological processes that attenuate or amplify impacts. Because of these interactions, even El Niño events considered to be a ‘non-event’ nationally can spell disaster for certain people and places. Recognising vulnerability as embedded in specific social-ecological systems highlights the need to combine El Niño-specific interventions with those that build general capacities to deal with multiple social and biophysical stresses and shocks. Thus, while there remains a need to invest in climate resilience programmes, such as early warning systems and weather insurance, building resilience to El Niño events and other climate extremes will require addressing development deficiencies in, for example, healthcare, sanitation, water supply and community institutions. Because climate change is likely to further amplify existing vulnerabilities and create new ones, building general and specific capacities across scales will become increasingly necessary to protect the most vulnerable in society from serious harm.

Evidence from this study, therefore, supports the need for integrated social-ecological systems analysis for understanding vulnerability to climate variability and the need to integrate climate resilience programming into wider development processes to address underlying causes of vulnerability to climate extremes.

## Electronic supplementary material

Below is the link to the electronic supplementary material.

Supplementary material 1 (PDF 20 kb)

## Footnotes

1 The Sustainable Poverty Alleviation from Coastal Ecosystem Services project (SPACES) aims to study the relationship between coastal ecosystems in Kenya and Mozambique and the wellbeing of poor people living along the coast (www.espa-spaces.org).

2 The Coastal Ecosystem Services in East Africa (CESEA) project is a collaboration between scientists and researchers in Tanzania, Kenya and the UK to find new ways to help local people maintain their coastal resources whilst escaping poverty (www.espa.ac.uk/projects/ne-l001535-1).

3 The Oceanic Niño Index (ONI) is the primary indicator for monitoring El Niño and La Niña. The ONI tracks the rolling 3-month average sea-surface temperatures in an area between 120°–170°W in east-central tropical Pacific, known as Niño 3.4 region.

4 Figure 7 probably represents an under-estimate of this decline, since it gives total catches rather than catch per unit effort (which was unavailable); given the 2.9% yr^−1^ population growth rate and increased productivity of fisheries technologies, it is highly probable that effort is increasing.
