# Supplementary material for: Multiple impact pathways of the 2015–2016 El Niño in coastal Kenya
Source: Ambio. 2020 Mar 9;50(1):174–89. doi: 10.1007/s13280-020-01321-z (PMC7708579; doi:10.1007/s13280-020-01321-z)
Supplement: Supplementary file 1 — Supplementary material 1 (PDF 20 kb) [file 13280_2020_1321_MOESM1_ESM.pdf]

Ambio

Electronic Supplementary Material
This supplementary material has not been peer reviewed

Title: **Multiple impact pathways of the 2015–2016 El Niño in coastal Kenya**

Matt Fortnam, Molly Atkins, Katrina Brown, Tomas Chaigneau, Ankje Frouws, Kemyline Gwaro, Mark Huxham, James Kairo, Amon Kimeli, Bernard Kirui, Katy Sheen

Appendix S1: Summary of the mixed methods and datasets used in this study

Methods	Description	Data outputs
**Participatory vulnerability assessment workshop (FG1):** 12 participants representing fisher associations, women's groups, village leadership, community officers and traders		
Hazard assessment matrix	Participants identify the hazards (including every day, irregular and large-scale) facing households and the community, the frequency of their occurrence, and the degree of impact on wellbeing. Discussed hazard interactions, coping strategies and hazards caused by El Niño	Range, importance and interactions of different exposures<br>Impact on and sensitivity of wellbeing domains<br>Coping mechanisms
Seasonal and El Niño calendar	Seasonal calendar matrix was constructed, with months on one axis and hazards, vulnerabilities and capacities on the other. Differences between a typical year and the 2015-16 El Niño year were discussed.	Difficult times of year<br>Livelihood strategies<br>Trends in seasonal activities<br>Influence of El Niño on seasons
Historical timeline	Timeline of key hazards, events, and historic socio-economic and environmental changes. Discussed how the nature and impact of past El Niño episodes compared with recent El Niño	Past shocks and trends (exposures) changes in their intensity, behaviour and impacts<br>How community sensitivities, capacities have changed over time<br>History of El Niño impacts
Venn diagram	Diagram showing key stakeholders, their relative importance and the closeness of their relationship with the community. Discussed their role generally and in El Niño preparedness, response and recovery.	Ranking of importance of community and external organisations<br>Sources of information on El Niño and support during crises
Community walk and hazard map	Drew a map of the community and identified sites and households prone to or affected by hazards, and community resources. Walked to places of interest and interacted with community members	Contextual information<br>Where and who affected by hazards (exposure and sensitivity)<br>Resources for coping and community adaptations
**Household (HH) surveys and interviews**		
Flooding household survey (HHS1)	Census survey of 102 households in the flood prone villages of Vichigini, Jimbo and Jasini	Characteristics of households<br>Impacts of flood event on households<br>Coping and recovery mechanisms
Female headed household survey (HHS2)	Survey of 20 female headed households in a flood prone areas of Vanga sub-location in Jimbo, Jasini and Vanga town	Impacts of flood event on female headed households<br>Hazards that affect household<br>Coping and recovery mechanisms
El Niño household survey	Survey of 15 households to understand changes in wellbeing since a previous survey of the same households, and impacts, preparations and perceptions about El Niño. Most of these data are being used for a further study across several study sites	El Niño preparations
Key informant interviews	Semi-structured interviews were held with local and national government, village chiefs, humanitarian and development NGOs	Institutional and organisational context<br>Impacts of and responses to El Niño 2015-16 and the Vanga flood event
**Biophysical methods**		
Precipitation	Precipitation data were obtained from the 0.5^{o} grid resolution monthly GPCC land-based dataset (available 1891–2016)(Schneider 2011). Data were downloaded from https://www.esrl.noaa.gov/psd/data/gridded/data.gpcc.html. We note that results were similar when using the Climate Research Unit precipitation data set	Monthly precipitation for lower river Umba drainage basin, including mean (1891-2016), during very strong El Niño events (where Niño 3.4 index between December to February (DJF) is > 1.5) and very strong La Nina events (DJF is <-1.5)

	(Harris 2014), although it did not correlate as strongly with the Lunga Lunga rain gauge data. Lunga Lunga monthly rain gauge data, located at 4.55°S, 39.11°E; station ID :9439046; (available 1960-2002) was obtained from Kenya Meteorological Department. For characterizing ENSO events, the Niño 3.4 index data were used, calculated from the HadISST1 (Rayner et al. 2003) and downloaded from https://www.esrl.noaa.gov/psd/gcos_wgsp/Timeseries/Niño34/.	
Turbidity	Relative turbidity measures for Vanga Bay over 2014-2016 taken from Landsat 8 imagery (Badjeck et al. 2010). All available images – those without extensive cloud cover - from March, April and May in 2014, 2015 and 2016 were examined, using ACOlite software and the 645/859 nm setting from Dogliotti et al. (2015) with OLI 655/865 or MSI 664/865 bands.	Turbidity maps for the bay and statistical comparisons of mean turbidity measurements in March, April and May 2016 compared with earlier years
Mangrove growth	Monthly growth increments of 101 trees in ten permanent monitoring plots at Gazi Bay	Mean monthly growth increments in *A. marina* and *R. mucronata*
Mangrove sedimentation	Monthly elevation measurements from five permanent modified RSET plots at Gazi Bay	Monthly surface elevation measures
Seagrasses sedimentation	Monthly elevation measurements from five modified RSET plots at Gazi Bay	Monthly surface elevation measures
Coral bleaching survey	Rapid assessment bleaching surveys at 24 sites in Kenya	Percent coral cover bleached
**Secondary data**		
Social data (SD1)	Data collected and digitized on water borne disease admissions from health centres, and school attendance from secondary school	Indicators of impact of flood event on health and education
Biophysical data	Fish landing data from Vanga, taken from the Kenya Fisheries Department monthly landing data	Long term fisheries trend and evidence for impact during El Niño event
